# The (p)ppGpp synthetase Rsh promotes rifampicin tolerant persister cell formation in *Brucella abortus* by regulating the type II toxin-antitoxin module *mbcTA*

**DOI:** 10.3389/fmicb.2024.1395504

**Published:** 2024-05-22

**Authors:** Xiaofang Liu, Pingping Wang, Ningqiu Yuan, Yunyi Zhai, Yuanhao Yang, Mingyue Hao, Mingxing Zhang, Dong Zhou, Wei Liu, Yaping Jin, Aihua Wang

**Affiliations:** ^1^College of Veterinary Medicine, Northwest A&F University, Xianyang, China; ^2^Key Laboratory of Animal Biotechnology of the Ministry of Agriculture, Northwest A&F University, Xianyang, China; ^3^College of Animal Science and Technology, Northwest A&F University, Xianyang, China

**Keywords:** (p)ppGpp synthetase Rsh, persister cells, type II TA modules, *mbcTA*, rifampicin, ATP levels

## Abstract

Persister cells are transiently tolerant to antibiotics and are associated with recalcitrant chronic infections due to recolonization of host cells after antibiotic removal. *Brucella* spp. are facultative pathogens that establish intracellular infection cycles in host cells which results in chronic persistent infections. *Brucella abortus* forms multi-drug persister cells which are promoted by the (p)ppGpp synthetase Rsh during rifampicin exposure. Here, we confirmed that Rsh promoted persister cells formation in *B. abortus* stationary phase treated with rifampicin and enrofloxacin. Deletion of the gene for Rsh decreased persister cells level in the presence of these drugs in different growth phases. However, persister cells formation by deletion strain varied in different growth phases in the presence of other antibiotics. Rsh also was involved in persister cells formation during rifampicin treatment under certain stress conditions, including acidic conditions, exposure to PBS, and heat stress. Moreover, Rsh impacted persister cell levels during rifampicin or enrofloxacin treatment in RAW264.7 macrophages. Certain typeIItoxin-antitoxin modules were upregulated under various stress conditions in *B. abortus*. We established that Rsh positively regulated the type II toxin-antitoxin *mbcTA*. Moreover, rifampicin-tolerant persister cells formation was elevated and ATP levels were decreased when *mbcTA* promoter was overexpressed in Rsh deletion background in stationary phase. Our results establish that (p)ppGpp synthetase Rsh plays a key role in *B. abortus* persistence and may serve as a potent novel target in combination with rifampicin in the development of new therapeutic approaches and prevention strategies to treat chronic infections of *Brucella*.

## 1 Introduction

*Brucella* spp. are facultative intracellular pathogens that are important human and veterinary pathogens. The bacterium causes brucellosis which induce abortion and infertility in animal livestock (Roop et al., 2021). *Brucella* establishes an intracellular infection cycle in macrophages to achieve intracellular proliferation. *Brucella* is widespread in more than 170 countries and is transmitted indirectly to humans through inhalation of airborne agents or directly by interaction with infected animals or contaminated animal products or as an occupational hazard (Qureshi et al., 2023). Human brucellosis is one of the world’s dominant pervasive zoonotic diseases (Guo et al., 2023). Although vaccine strains including *Brucella abortus* A19, *B. melitensis* M5, *B. abortus* S19 and *B. suis* S2, are used commonly to prevent and control brucellosis in livestock (He et al., 2022), these vaccines possess residual virulence which increases persistence and causes infectious risk in animals and humans during vaccination (He et al., 2022). The choice of antibiotic for treatment of human brucellosis is restricted (Ficht, 2003), although doxycycline and rifampicin are used most frequently (Qureshi et al., 2023). In addition, intricate contacts between bacteria and host may establish chronic infection and long-term survival in hosts in certain situations (He et al., 2022). First, the bacteria may evade host immune responses to achieve long-lasting infection. Second, the emergence of antimicrobial resistance in *Brucella* allows the pathogen to survive treatment (Qureshi et al., 2023). Third, bacteria may evade killing from usually lethal doses of antibiotic by entering a physiologically dormant state referred to as persistence (Maisonneuve and Gerdes, 2014; Mode et al., 2022). Persister cells may lead to relapse of infection after removal of antibiotic treatment and thus are implicated in antibiotic treatment failure (Fisher et al., 2017). Persister cells in *B. abortus* may form experimentally upon multi-antibiotic treatment either in infected cells or when growing in culture medium (Mode et al., 2022; Liu et al., 2023). Understanding the latent mechanisms by which *B. abortus* forms persister cells and elucidating the mechanisms that are involved are important for effective disease control and prevention of brucellosis.

Bacterial populations are exposed constantly to change and stressful environmental conditions which require adaptation for survival (Rizvanovic et al., 2022). Persister cells are a subgroup of the population that display transient antibiotic tolerance and phenotypic heterogeneity along with diverse growth and survival characteristics due to changes in gene expression (Maisonneuve and Gerdes, 2014; Fisher et al., 2017). Persister cells are characterized by a dormant or slowing-growth state, low metabolic activity, and an absence of changes in genetic heritability which together promote the temporary inactivation of antibiotic targets (Wilmaerts et al., 2019). Persister cells may resume growth after removal of antibiotic exposure, but remain antibiotic sensitive (Maisonneuve and Gerdes, 2014). Persister cells are mainly classified into type I and type II (Balaban et al., 2004). Type I persister cells are non-growing cells that generally form in stationary phase or in response to external triggers including antibiotic stress. Type II persister cells form randomly by phenotypic transition without external triggers and may switch back to a normal phenotype during growth (Balaban et al., 2004). Persister cells cause prolonged and recurrent infection which are one of the major public health concern (Stapels et al., 2018; Gollan et al., 2019; Kaldalu et al., 2020). Persistence has been observed widely in diverse bacteria, including *Salmonella* (Helaine et al., 2014; Cheverton et al., 2016; Stapels et al., 2018), *Escherichia coli* (Shan et al., 2017), *Mycobacterium tuberculosis* (Parbhoo et al., 2022), and *Staphylococcus aureus* (Peyrusson et al., 2022).

The alarmone (p)ppGpp typically is generated in response to nutrient starvation and other stresses to reprogram cellular physiology for maintaining bacterial growth, metabolic homeostasis, and survival functions (Steinchen et al., 2020). The (p)ppGpp molecule in *E. coli* is regulated by RelA and SpoT enzymes. Analogously, (p)ppGpp is synthesized by the RelA-SpoT homologue *Rsh* in *Brucella* (Hanna et al., 2013; Das and Bhadra, 2020). The stringent response that involves (p)ppGpp as a ubiquitous second messenger is essential in numerous bacterial infectious processes (Maisonneuve and Gerdes, 2014), and (p)ppGpp also is required for virulence, persistence, and antimicrobial resistance (Das and Bhadra, 2020; Pacios et al., 2020). It was shown previously that Rsh facilitates persister cell formation in *B. abortus* in the presence of rifampicin (Pacios et al., 2020; Liu et al., 2023). The relationship between Rsh and persistence also was demonstrated in other bacteria, including *E. coli* (Svenningsen et al., 2019) and *Pseudomonas aeruginosa* (Martins et al., 2018).

The (p)ppGpp alarmone and toxin-antitoxin (TA) modules are tightly intertwined during persister cell formation (Prax and Bertram, 2014; Eisenreich et al., 2020). Nevertheless, certain TA systems are up-regulated under stress and participate in persister cell production independently of (p)ppGpp (Jurėnas et al., 2022). TA genes typically are operons that encode a toxin that interferes with cellular processes and an antitoxin that inhibits the homologous toxin (Fisher et al., 2017; Jurėnas et al., 2022). TA systems are divided into different types among which types I and II allow bacterial populations to adapt to stressful conditions and participate in persister cell formation (Harms et al., 2016; Jurėnas et al., 2022). Nevertheless, there are contradictory observations concerning the role of TA systems and persistence in different bacterial species and different experimental setups (Pacios et al., 2020). Moreover, the relationship between (p)ppGpp and TA systems in *Brucella* persister cells remains poorly understood.

As the (p)ppGpp synthetase Rsh was linked to persister cell formation after rifampicin exposure (Liu et al., 2023), here we sought to investigate the underlying mechanism by which Rsh influences persistence in the stationary phase in *B. abortus*. We demonstrate that persister cell production involving Rsh is related to growth phase and antibiotic classes in *B. abortus*. Specifically, Rsh only stimulated persister cell formation in different growth phases only during rifampicin or enrofloxacin treatment among the antibiotics that were tested. Rsh-mediated persistence also was observed under different stress conditions and in RAW264.7 macrophage cells. Certain type II TA modules in *B. abortus* were upregulated under stress conditions. We show that Rsh contributed to persister cell formation by positively regulating the type II TA MbcTA in *B. abortus* stationary phase after rifampicin exposure. Moreover, ATP levels were elevated when the *mbcTA* promoter was overexpressed in stationary phase in a Rsh deletion background. These data provide new clues for discovering potential drug targets for clearing *Brucella* persister cells, as well as for forming new strategies for prevention of *Brucella* infection.

## 2 Results

### 2.1 Rsh promotes persister cell formation in the presence of rifampicin and enrofloxacin in *Brucella abortus* stationary phase

**Figure 1:**
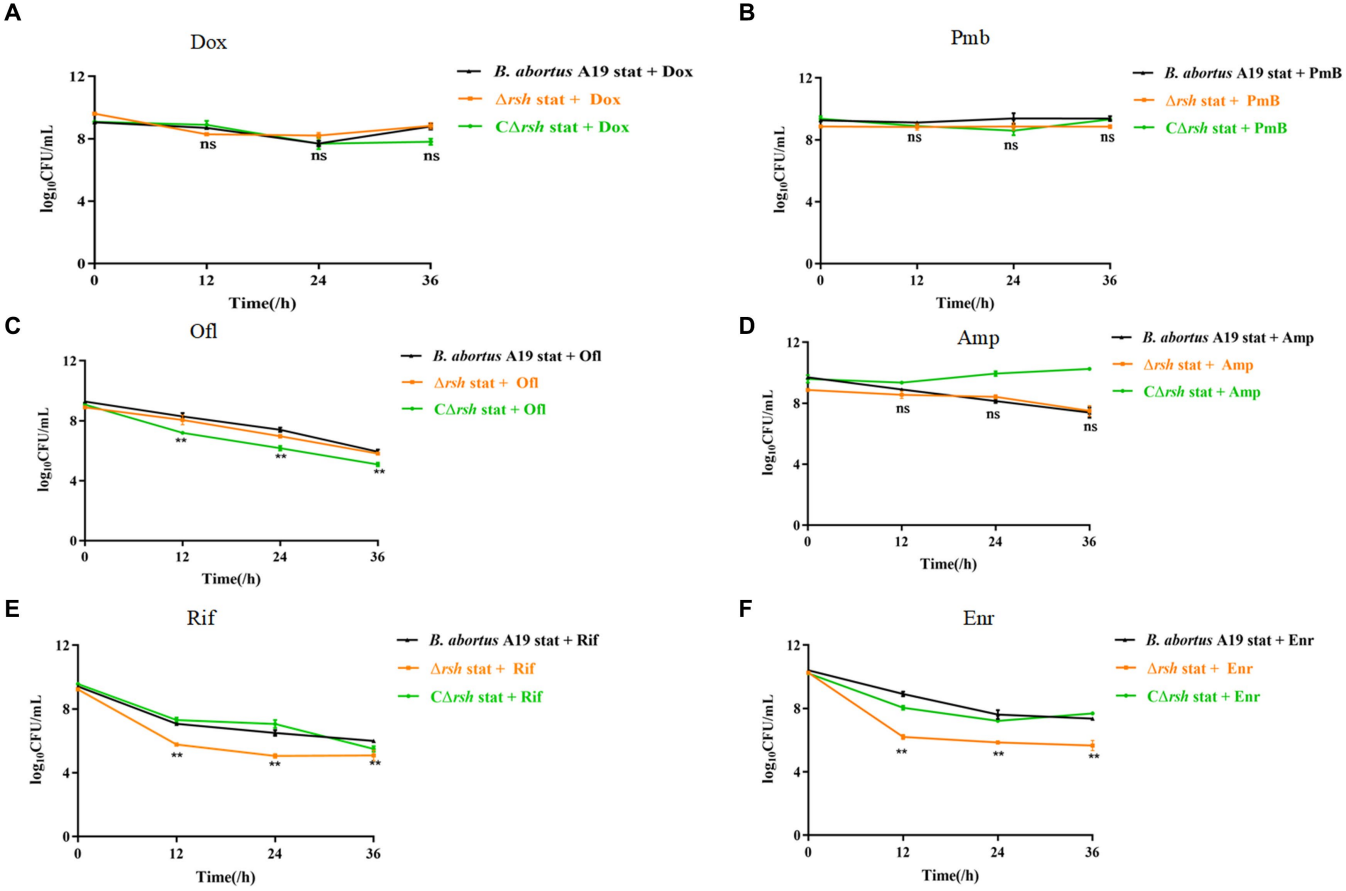
Rsh influences persister cell formation in *B. abortus* stationary phase during rifampicin and enrofloxacin exposure. **(A)** Doxycycline (Dox) (1.2 μg/mL); **(B)** Polymyxin B (40 μg/mL); **(C)** Ofloxacin (40 μg/mL); **(D)** Ampicillin (8 μg/mL); **(E)** Rifampicin (40 μg/mL); **(F)** Enrofloxacin (12 μg/ mL). The strains were grown to late stationary phase (72 h) in TSB medium. The cultures were treated with antibiotics for an additional 36 h to detect persister cells. The cultures were washed once in PBS and viable counts were enumerated by plating 10-fold serial dilutions onto TSB agar medium. All experiments were repeated independently three times. Data are analysed with two-way ANOVA. Data are mean ± S.D. Asterisks indicate statistically significant differences: * *p <* 0.05; ** *p* < 0.01.

The relationships between the stringent response alarmone (p)ppGpp and persistence are dissimilar in different bacteria (Conlon et al., 2016; VandenBerg et al., 2016; Pacios et al., 2020). Previous research from our laboratory indicated that *B. abortus* was capable of forming multi-drug tolerant persister cells (Liu et al., 2023)*. B. abortus* displayed a biphasic killing curve with a subpopulation of surviving persister cells during different antibiotic treatment, except with gentamicin. Moreover, deletion of the gene for Rsh (Δ*rsh*) resulted in decreased persistence in either exponential phase or stationary phase with rifampicin treatment for 24 h (Liu et al., 2023). Therefore, we examined whether Rsh promoted persister cell production in *B. abortus* stationary phase during different antibiotic treatments for 36 h. Deletion of the gene for Rsh had no effect on persister cell formation in stationary phase in the presence of doxycycline (Figure 1A), polymyxin B (Figure 1B), ofloxacin (Figure 1C), or ampicillin (Figure 1D) compared with wild-type *B. abortus*. However, persister cell formation in stationary phase during ofloxacin treatment was reduced in the complemented strain (CΔ*rsh*) compared with wild-type or Δ*rsh* strains (*p < 0.01*) (Figure 1C). Moreover, the deletion strain showed significantly reduced persister cell levels in stationary phase during rifampicin and enrofloxacin treatment for 36 h (*p < 0.01*) (Figures 1E,F). These results indicate that Rsh participated in persister cell formation in *B. abortus* stationary phase during rifampicin and enrofloxacin treatment, but not when other antibiotic classes were tested.

### 2.2 The role of Rsh in persister cell formation is linked to both growth phase of *Brucella abortus* and antibiotic classes

**Figure 2:**
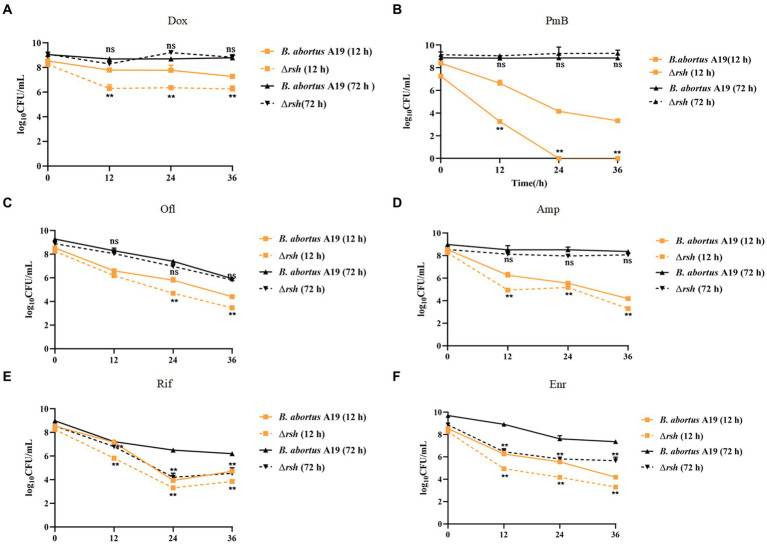
Relationship of Rsh and persister cell formation to growth phase and antibiotic types. **(A)** Doxycycline (1.2 μg/mL); **(B)** Polymyxin B (40 μg/mL); **(C)** Ofloxacin (40 μg/mL); **(D)** Ampicillin (8 μg/mL); **(E)** Rifampicin (40 μg/mL); **(F)** Enrofloxacin (12 μg/ mL). *B. abortus* strains were grown to exponential phase (12 h), or late stationary phase (72 h) in TSB medium. Cultures then were treated with antibiotics for additional 36 h to detect persister cell formation. Treated cultures were washed once in PBS and viable counts were enumerated at the indicated times by plating 10-fold serial dilutions onto TSB agar. All experiments were repeated independently three times. Data are mean ± S.D. Data are analysed with two-way text. Asterisks indicate statistically significant differences: * *p <* 0.05; ** *p* < 0.01.

We showed previously that persistence in *Brucella* was growth phase-dependent, by assessing exponential (12 h) and late stationary (72 h) phases during exposure to different antibiotic classes. These findings led us to assess whether Rsh affects persister cell production during different growth phases with different antibiotic treatments. Here, we used an analogous approach to investigate the extent to which Rsh impacted persistence in different growth phases following treatment with different antibiotics for 36 h. Wild-type and Δ*rsh* strains were grown to exponential (12 h) and late stationary (72 h) phases (Liu et al., 2023). Aliquots were removed and cultured in TSB medium containing different antibiotics for 36 h. Similar with wild type, we found Rsh formed the time-dependent biphasic kill curves. There was a significant decrease in persister cells in Δ*rsh* background compared with wild-type after doxycycline treatment in exponential phase (*p* < 0.01) (Figures 2A–D). However, no change in persister cell levels in the Δ*rsh* mutant was detected in stationary phase (Figures 2A–D). After rifampicin or enrofloxacin treatment, persister cell formation in the Δ*rsh* mutant was significantly lower than wild-type in both exponential and stationary phases (*p < 0.01*) (Figures 2E,F). In summary, we observed that persister cell formation by *B. abortus* was growth phase-dependent in both wild-type and Δ*rsh* strains with different antibiotic treatments. These data suggest that persister cell production linked to Rsh depends both on growth phase and on antibiotic classes in *B. abortus*.

### 2.3 Changes in persister cell formation in the *Δrsh* mutant are not due to enhanced susceptibility to rifampin and enrofloxacin

**Figure 3:**
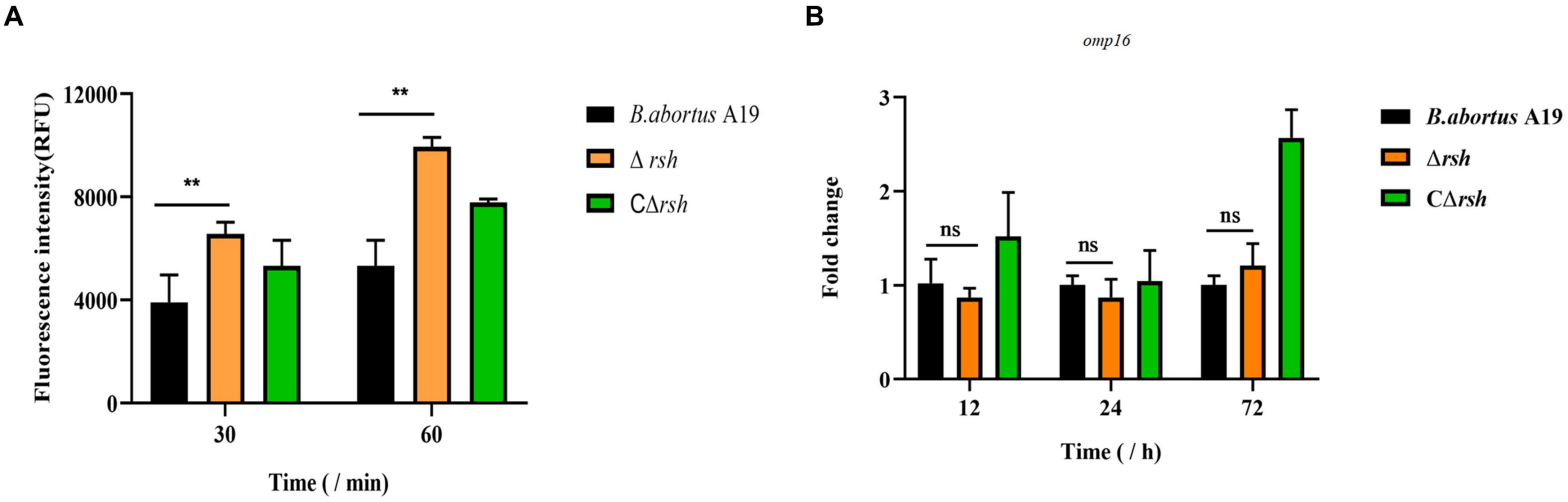
Permeability of cell wall of *B. abortus* Δ*rsh* in stationary phase. **(A)** Ethidium bromide uptake by Δ*rsh* strain in stationary phase. **(B)** The *omp*16 mRNA levels in Δ*rsh* mutant background. Ethidium bromide was added to a final concentration of 2 μg/mL. Uptake was measured using 96-well microplates with excitation at 544 nm and emission at 590 nm. RNA was extracted and quantified using qRT-PCR. Each sample is normalized to 16S rRNA. Data are mean ± S.D. Data are analysed with one-way ANOVA; * *p* < 0.05; ** *p* < 0.01.

To assess whether reduced persister cell formation by the Δ*rsh* strain was due to decreased sensitivity to antibiotics, we determined MICs of the mutant for rifampicin and enrofloxacin. The MIC values for wild-type, Δ*rsh,* and CΔ*rsh* strains were similar for both drugs (Table 1) which suggests that the persister cell defect in Δ*rsh* is not due to increased antibiotic resistance. As rifampicin enters by diffusion through the cell wall (Namugenyi et al., 2017), we tested cell envelope permeability of the Δ*rsh* mutant by ethidium bromide uptake assays. The Δ*rsh* strain showed significantly elevated levels of ethidium bromide uptake relative to wild-type or CΔ*rsh* strains (*p* < 0.01) (Figure 3A). The outer membrane protein Omp16 plays an important role in maintenance of cell wall integrity of *Brucella* (Zhi et al., 2020). Therefore, we tested expression of *omp*16 in wild-type, Δ*rsh* and CΔ*rsh* strains using qRT-PCR. Expression of *omp*16 was similar in the three strains in different growth phases (Figure 3B). Overall, these data suggest that reduced persister cell by Δ*rsh* derivative is not due to enhanced susceptibility to rifampicin and enrofloxacin, but may be due to elevated cell envelope permeability which is unrelated to the Omp16 protein.

**Table 1:** MIC determination of *B. abortus* A19 strains.^a^

Antibiotics	Rifampicin (Rif)	Enrofloxacin (Enr)
*B. abortus* A19	1.95	1.20
*∆rsh*	1.95	1.20
*C∆rsh*	1.95	1.20

a MICs of the above antibiotics (μg/mL) were detected by using serial two-fold antibiotic dilutions in TSB broth.

### 2.4 Rsh plays a key role in persister cell formation during diverse stresses

**Figure 4:**
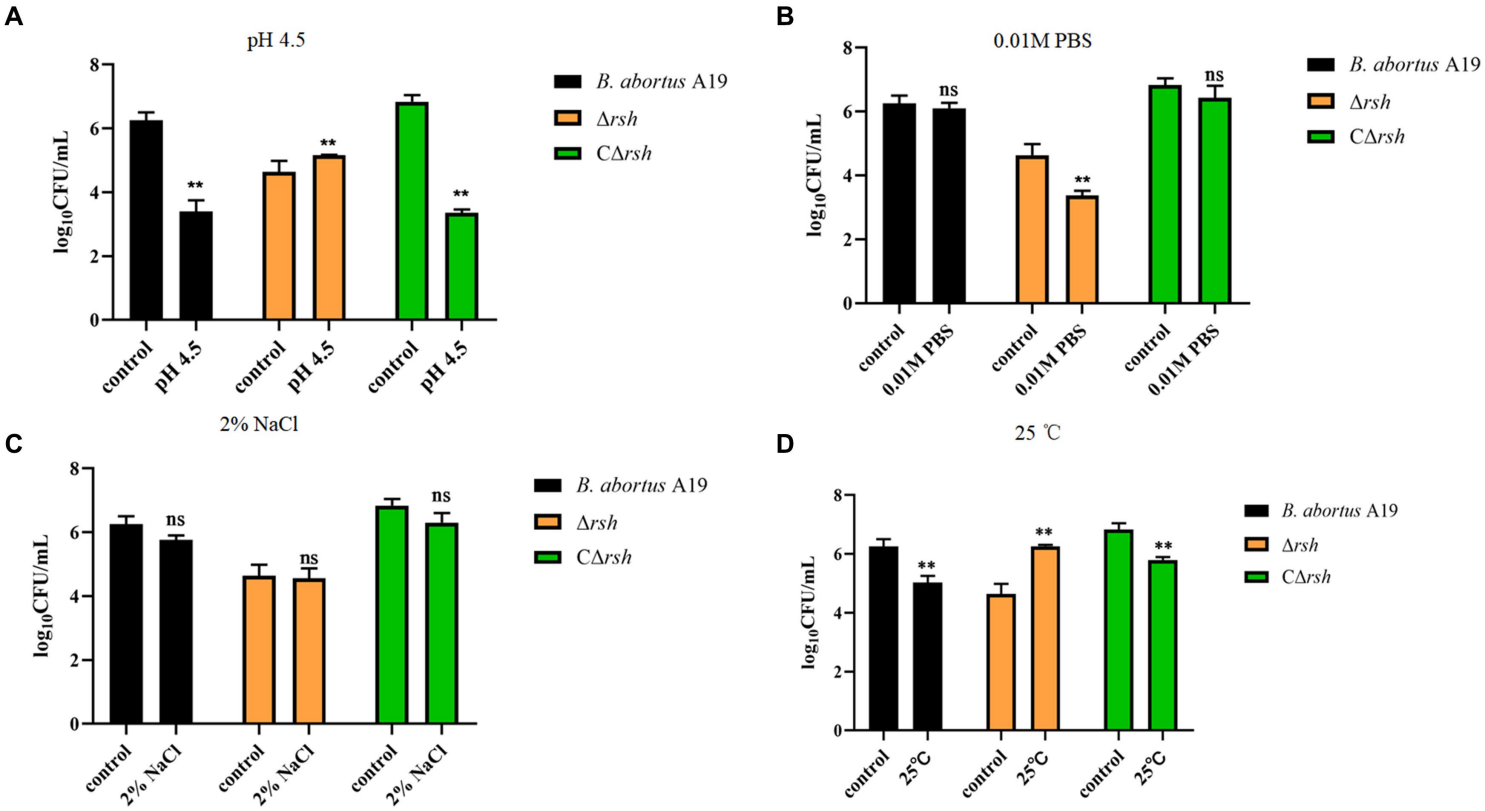
Persister cell formation by *B. abortus* Δ*rsh* after exposure to environmental stressors. **(A)** pH 4.5. **(B)** 0. 01 M phosphate buffer. **(C)** 2% NaCl. **(D)** 25°C. Wild-type *B. abortus* A19, Δ*rsh* and CΔ*rsh* stationary phase cells (1–2 × 10^8^ CFU/mL) were exposed to environmental stress conditions for 1 h and were then treated with rifampicin (40 μg/mL) for 24 h. Data are mean ± S.D. Data are analysed with one-way ANOVA;* *p* < 0.05; ** *p* < 0.01.

We next examined whether environmental stresses induced Rsh-mediated persister cell formation in the stationary phase in *B. abortus*. Survival of Δ*rsh* decreased significantly compared with wild-type or CΔ*rsh* strains after exposure to acid, phosphate, NaCl, or heat stress for 1 h (Figures 4A–D). Moreover, the absence of *rsh* increased persister cell levels during acid and heat stress after rifampicin treatment for 24 h compared with wild-type or CΔ*rsh* strains (Figures 4A,D). The Δ*rsh* mutant also displayed significantly decreased persister cell production during phosphate stress after rifampicin treatment compared with wild-type or complemented strains (Figure 4B). No differences in persister cell levels were observed for the three strains during NaCl stress (Figure 4C). The results suggest that Rsh plays an important role in certain stress-induced persister cell formation in the stationary phase in *B. abortus*.

### 2.5 Rsh promotes persistence in RAW264.7 macrophages after rifampicin and enrofloxacin exposure

**Figure 5:**
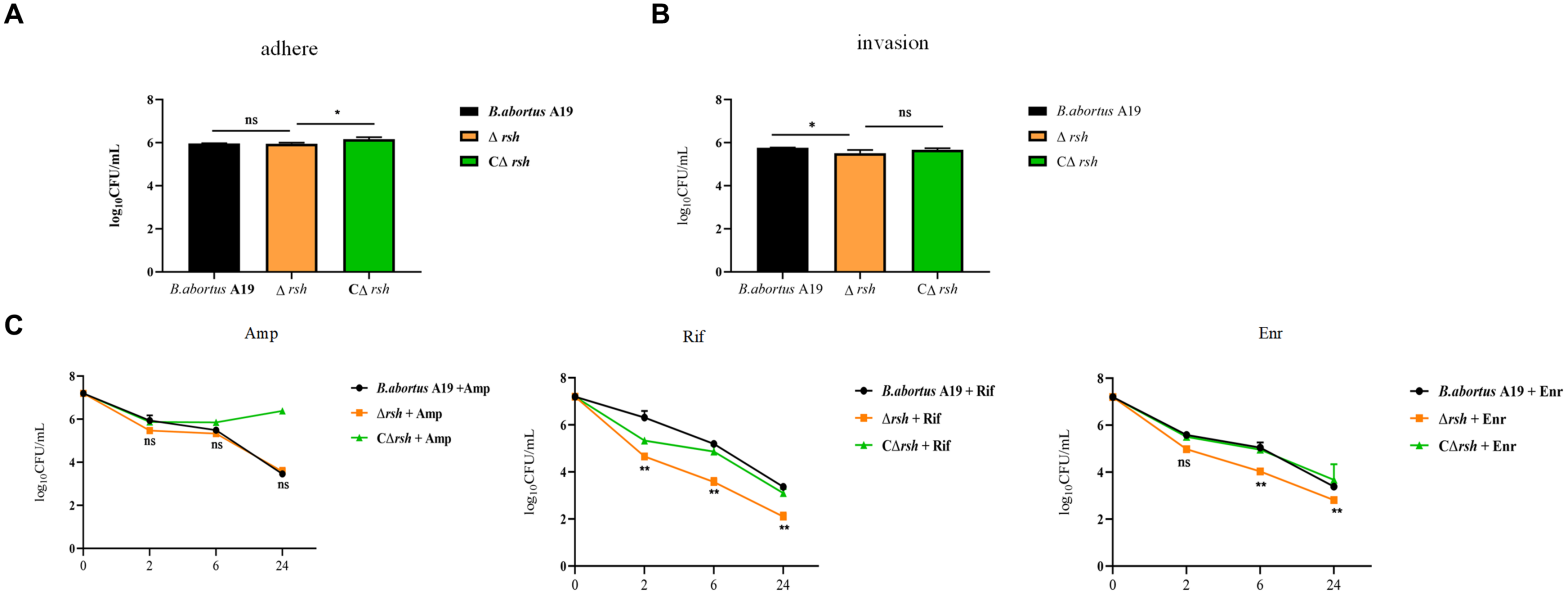
The effects of (p)ppGpp synthetase Rsh on adhesion, invasion, and persistence in RAW264.7 macrophages. **(A)** Adherence of Δ*rsh* in RAW264.7 cells. **(B)** Invasion of Δ*rsh* in RAW264.7 cells. **(C)** Persister cell formation by Δ*rsh* in RAW264.7 cells with ampicillin, rifampicin, and enrofloxacin treatment. Adhesion of bacteria to RAW264.7 cells was allowed to occur for 1 h. Invasion of bacteria in macrophage cells was performed by adding gentamycin (50 μg/mL) to the medium and incubating for an additional 1 h. The cultures were co-incubated with RAW264.7 cells for 1 h and then grown in TSB medium containing ampicillin (4 μg/mL), rifampicin (20 μg/mL), or enrofloxacin (6 μg/mL) for testing persister cell levels. Data are mean ± S.D. Data are analysed with one-way or two-way ANOVA; * *p* < 0.05; ** *p* < 0.01.

*Brucella* is an intracellular pathogen that produces persister cells following internalization by macrophages (Mode et al., 2022; Liu et al., 2023). To investigate whether Rsh is also involved in persistence in macrophages, we first utilized RAW264.7 cells to investigate the role of Rsh on adhesion and invasion by *B. abortus*. Wild-type, Δ*rsh* and CΔ*rsh* strains showed similar adhesion properties to RAW264.7 cells (Figure 5A). However, invasion by Δ*rsh* mutant was less compared with wild-type and CΔ*rsh* strains (*p* < 0.05) (Figure 5B). We next investigated persister cell formation by Δ*rsh* mutant in RAW264.7 cells after ampicillin, rifampicin, or enrofloxacin treatment for 24 h. Similar to our preceding results *in vitro*, there was no difference in persister cell formation by the mutant strain after ampicillin treatment compared with wild-type strain. In contrast, persister cell levels of Δ*rsh* mutant were reduced significantly in RAW264.7 cells compared with wild-type strain in the presence of rifampicin and enrofloxacin (Figure 5C). These results show that Rsh influences *B. abortus* persistence in macrophages in the presence of rifampicin and enrofloxacin.

### 2.6 Type II TA modules in *Brucella abortus* are regulated by environmental stresses

**Figure 6:**
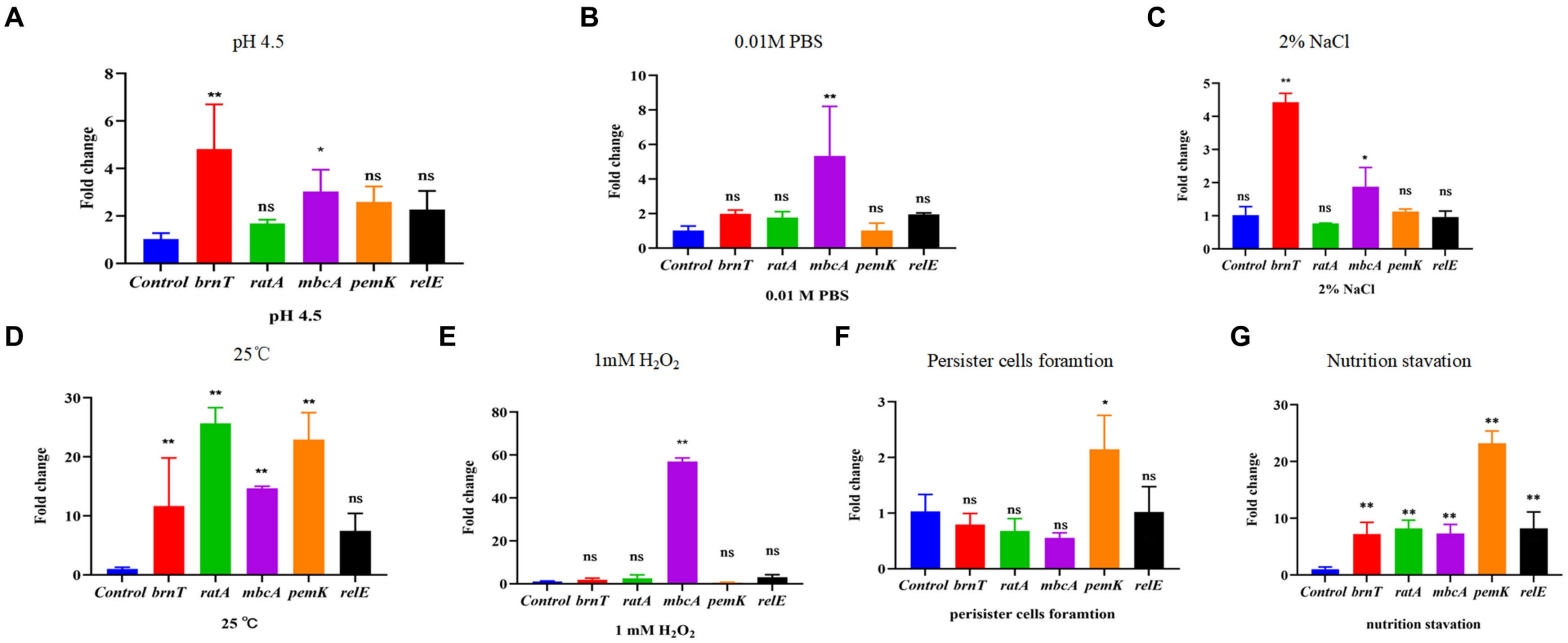
Certain type II TA genes in *B. abortus* are induced under environmental stress conditions. **(A)** HCl (pH 4.5). **(B)** 0.01 M phosphate buffer (pH 7.0). **(C)** 2% NaCl. **(D)** 25°C. **(E)** 1 mM H_2_O_2_. **(F)** Nutrition starvation. **(G)** Persister cell formation. Stationary-phase cultures were exposed to the indicated stress conditions and mRNA levels of type II TA genes were quantified using qRT-PCR. Each sample is normalized to 16S rRNA. Data are mean ± S.D. Data are analysed with one-way ANOVA; * *p* < 0.05; ** *p* < 0.01.

Rsh is up-regulated in *B. abortus* A19 under various stress conditions (Liu et al., 2023). Type II TA systems also regulate stress reactions and persister cell production during antibiotic exposure (Lobato-Márquez et al., 2016). Certain TA modules are expressed during nutrient starvation which induces persister cell formation (Zhou et al., 2021). We screened known and predicted type II TA modules in *B. abortus* by RT-qPCR for induction of transcription by various treatment including acid, phosphate, NaCl, temperature, H_2_O_2_ exposure, persister cells formation and nutrition starvation. Low pH and NaCl stress increased transcription of the *brnT* (*p < 0.01*) and *mbcA* (*p < 0.05*) TA modules, whereas other TA genes showed no changes (Figures 6A,C). Moreover, expression of *mbcA* also was elevated >4-fold under phosphate and H_2_O_2_ stresses (*p < 0.01*) (Figures 6B,E). Expression of other TA modules genes did not vary significantly under these conditions. Interestingly, TA genes were up-regulated (*p* < 0.01) during temperature stress, except for *relE* locus (Figure 6D). The *pemK* was significantly increased (*p* < 0.01) during persister cells formation (Figure 6F). And all gene were up-regulated (*p* < 0.01) during nutrition starvation (Figure 6G). These results indicate that some, but not all, type II TA modules are induced under stress conditions, persister cells formation and nutrition starvation in stationary phase in *B. abortus*.

### 2.7 Stationary phase expression of type II TA modules is altered by *rsh* deletion in *Brucella abortus*

**Figure 7:**
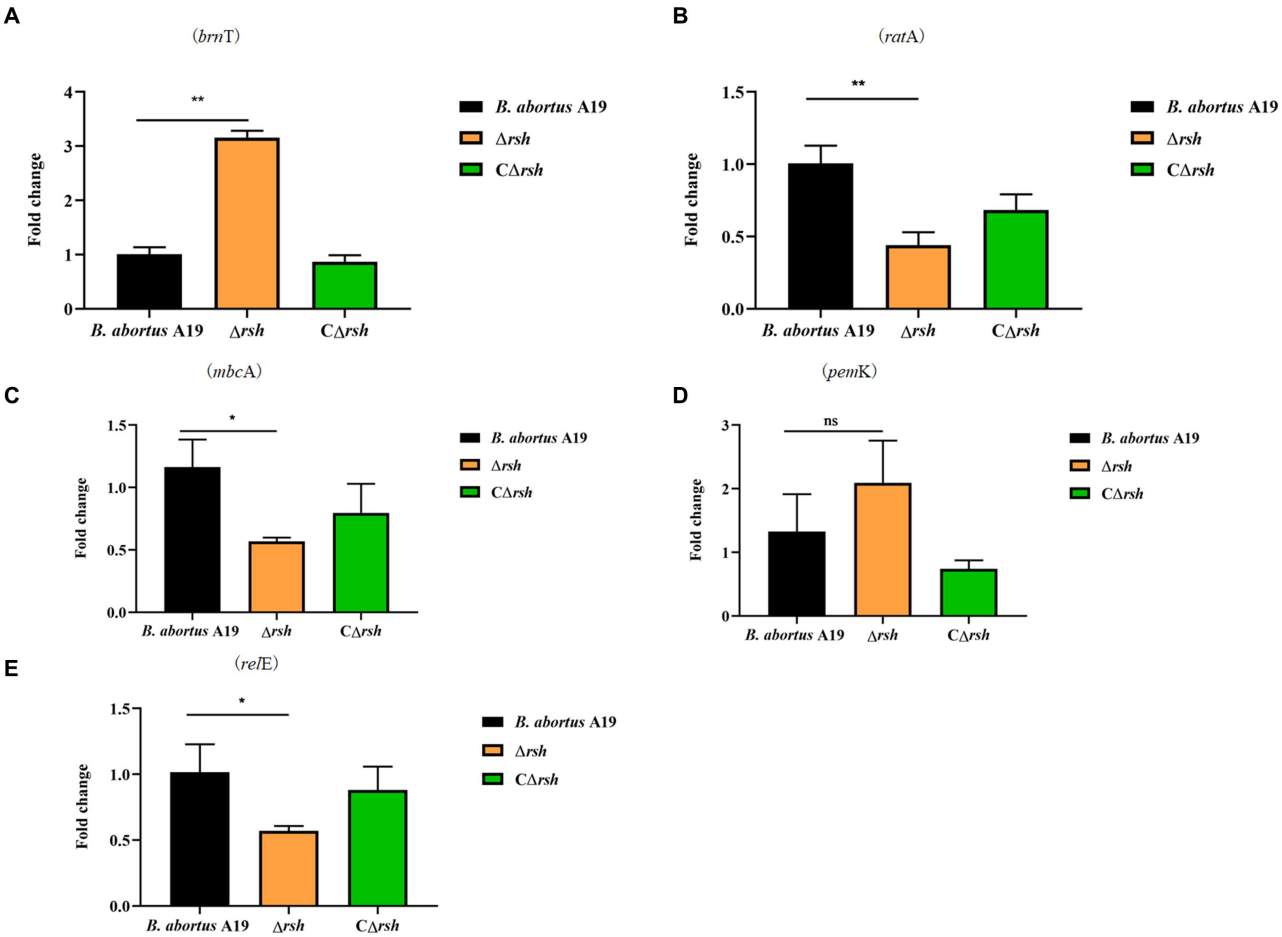
Expression level of type II TA genes in the Δ*rsh* deletion background in stationary phase. **(A)** *brnT*. **(B)** *ratA*. **(C)** *mbcA*. **(D)** *pemK*. **(E)** *relE*. RNA of cultures in stationary phase was extracted and mRNA levels of type II TA genes were quantified by qRT-PCR. Each sample is normalized to 16S rRNA. Data are the means ± S.D. Data are analysed with one-way ANOVA; * *p* < 0.05; ** *p* < 0.01.

The levels of alarmone (p)ppGpp depend on nutrient availability as well as other parameters, including oxygen concentration, pH, osmotic shock, temperature shift, and environmental stresses (Pacios et al., 2020). As the *hipT* induces (p)ppGpp synthesis in *E. coli* (Vang Nielsen et al., 2019), we assessed whether there also was a link between (p)ppGpp and type II TA modules in stationary phase cells of *B. abortus*. Expression of five TA modules was detected by qRT-PCR in the Δ*rsh* mutant in stationary phase. Moreover, the *brnT* type II TA module was up-regulated significantly in the mutant compared with wild-type or CΔ*rsh* strains (Figure 7A). In contrast, expression of the *mbcA*, *ratA* and *relE* loci decreased in the Δ*rsh* strain (Figures 7B,C,E), but expression of *pemK* was unchanged (Figure 7D). Our results demonstrate that there may be regulatory links between Rsh and certain type II TA modules in *B. abortus*.

### 2.8 Rsh promotes persister cell formation by positively regulating *mbcTA* in *Brucella abortus* stationary phase

**Figure 8:**
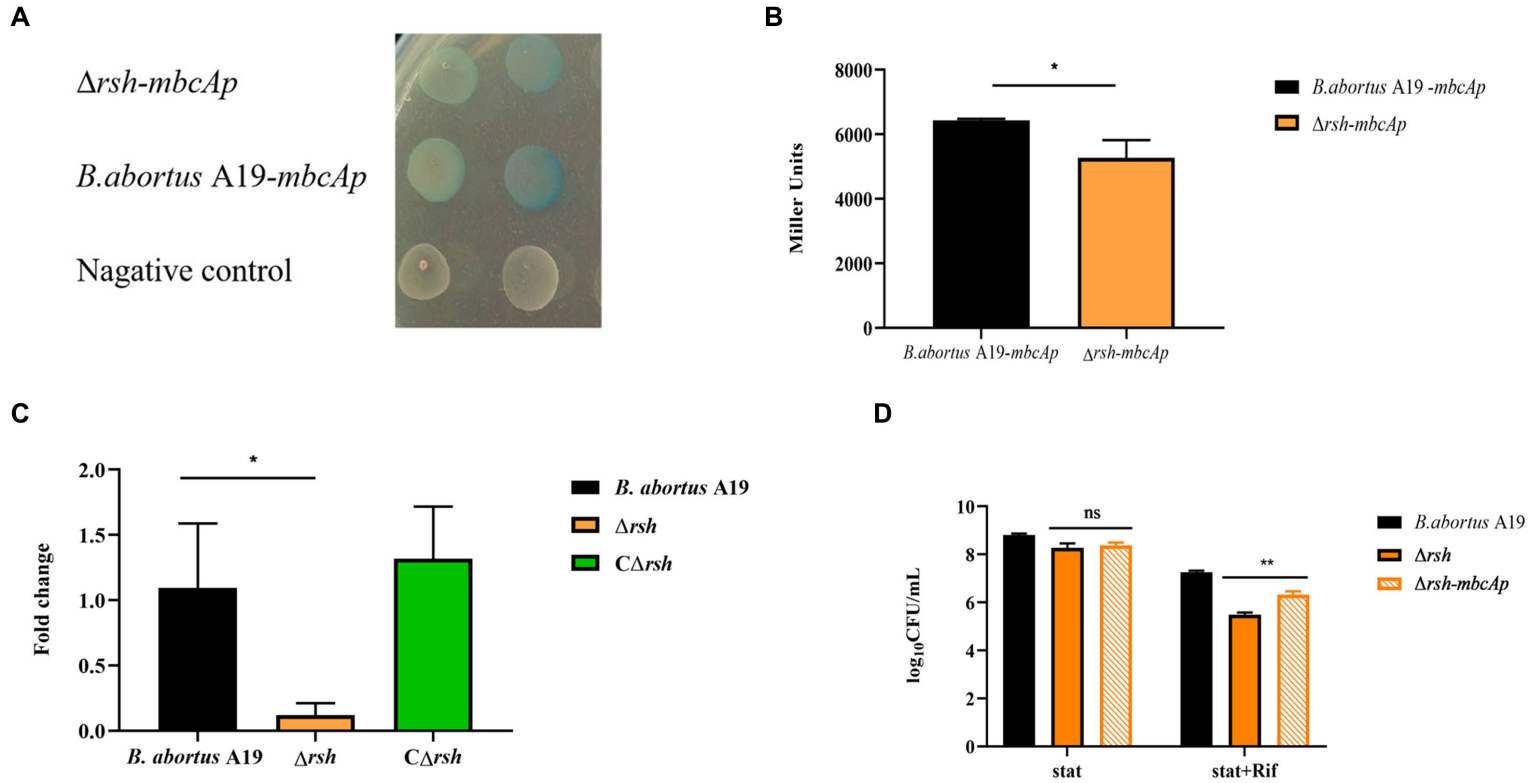
Rsh promotes persister formation by regulating *mbcTA* after rifampicin exposure in *B. abortus* stationary phase. **(A)** Color reaction with X-gal of wild-type and Δ*rsh* strains carrying a pBB-*mbcTAp*-*lacZ* promoter fusion plasmid. **(B)** β-galactosidase assay of wild-type and Δ*rsh* strains carrying a pBB-*mbcTAp*-*lacZ* promoter fusion plasmid. **(C)** The mRNA levels from the *mbcTA* promoter in wild-type and Δ*rsh* strains. **(D)** Persister cell formation of wild-type and Δ*rsh* strains with pBB- *mbcTAp*-*lacZ* promoter fusion plasmid. Wild-type and Δ*rsh* strains carrying a pBB- *mbcTAp*-*lacZ* promoter fusion plasmids were cultured to stationary phase in TSB medium. The stationary-phase cultures were spotted onto TSB agar with X-gal and tested by β-galactosidase assay. The RNA of stationary-phase cultures was extracted and mRNA was quantified by qRT-PCR. Each sample is normalized to 16S rRNA. Data are mean ± S.D. Data are analysed with one-way ANOVA; * *p* < 0.05; ** *p* < 0.01.

As expression of the *mbcTA* and *brnT* TA genes was altered in the Δ*rsh* strain, we assessed whether these changes were of functional relevance. The *brnTA* promoter is located in the upstream region (543 bp) of the *brnT* gene (Heaton et al., 2012). We constructed pBB-*brnTAp*-lacZ and pBB-*mbcTAp*-*lac*Z reporter fusion plasmids that contained the upstream region (343 bp) of *brnTA* and the putative promoter of *mbcTA* (312 bp), respectively, fused to *lacZ* transcriptional reporter gene (Supplementary Figure S1). The reporter plasmids were transformed into wild-type *B. abortus* A19 and Δ*rsh* strains. Promoter activity was detected by blue-white selection on plates with X-gal and by β-galactosidase assays. Expression of *mbcTA* promoter in wild-type and Δ*rsh* strains generated blue colonies in contrast to the negative control which produced white colonies (Figure 8A). Assays of β-galactosidase activity showed that *mbcTA* promoter was expressed more strongly in wild-type background compared with Δ*rsh* strain (Figure 8B) (*p* < 0.05). RT-qPCR confirmed that expression of *mbcTA* promoter was weaker in mutant strain than wild-type (Figure 8C). In contrast, the activity of *brnTA* promoter was not detected in either β-galactosidase or blue-white assays (data not shown). The additional 200 bp upstream of *brnTA* gene that is missing in the reporter used here may be important for promoter activity (Heaton et al., 2012). In summary, our results indicate that Rsh positively regulates *mbcTA* promoter in *B. abortus*.

To examine whether Rsh accelerated persister cell formation by regulating the *mbcTA* promoter in stationary phase. The wild-type, ∆*rsh* and ∆*rsh* with pBB-*mbcTAp*-*lac*Z reporter plasmid (∆*rsh-mbcTAp*) strains were cultured in stationary phase for 72 h and then were treated with rifampicin for 24 h to induce persister cells. Persister cell numbers increased significantly in ∆*rsh*-*mbcTAp* strain compared with ∆*rsh* strain (Figure 8D) which indicate that Rsh promotes persister cells formation by positively regulating *mbcTA* in *B. abortus* stationary phase after rifampicin treatment.

### 2.9 The (p)ppGpp synthetase Rsh decreases ATP levels in *Brucella abortus* stationary phase

**Figure 9:**
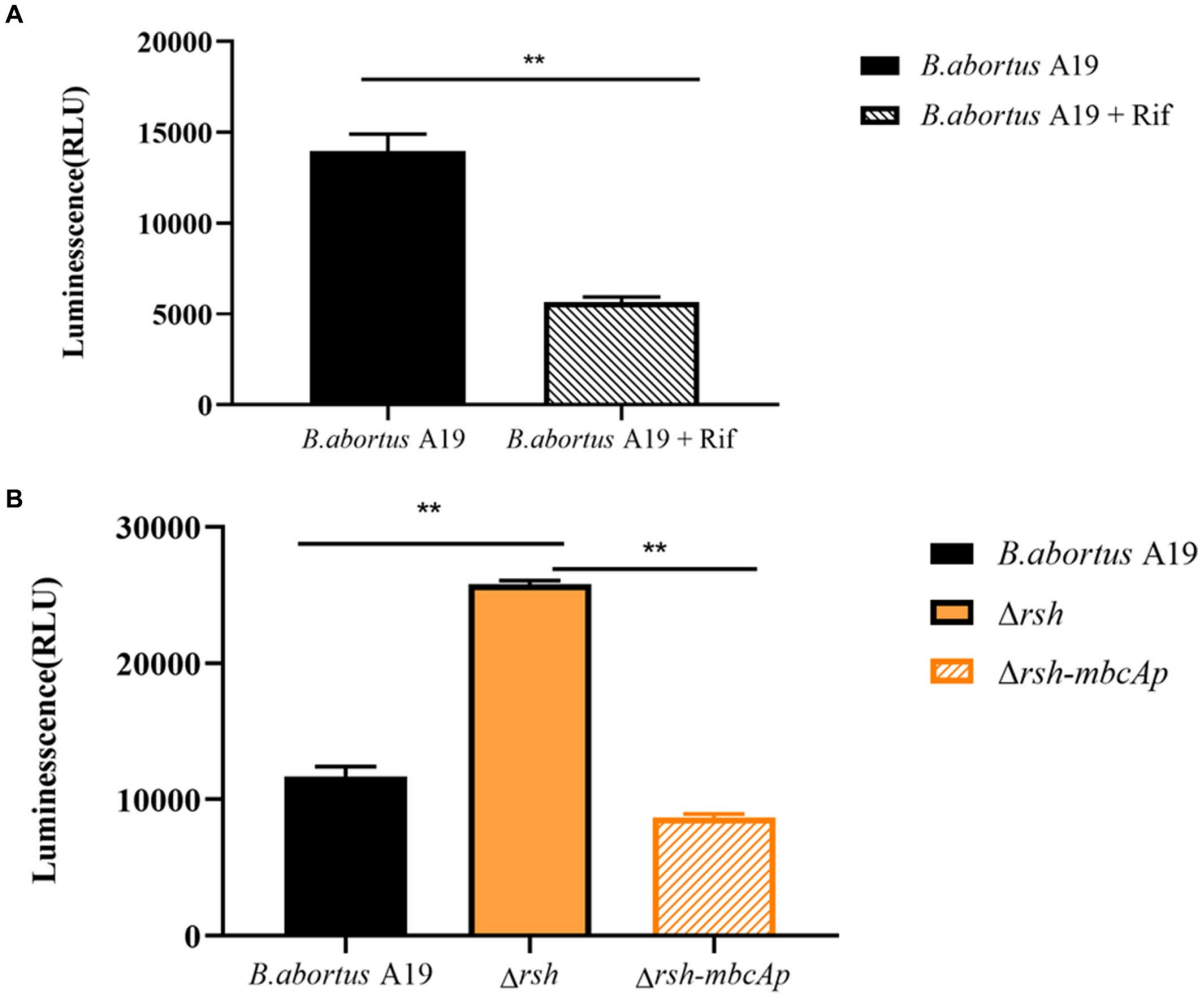
ATP levels of *B. abortus* rifampicin-tolerant persister cells, ∆*rsh* and ∆*rsh-mbcTAp* strains in stationary phase. **(A)** ATP levels of rifampicin-tolerant stationary phase persister cells of *B. abortus.* **(B)** ATP levels of ∆*rsh*-*mbcTAp* in stationary phase. The wild-type, ∆*rsh* and ∆*rsh*-*mbcTAp* strains were grown to stationary phase for 72 h. The stationary phase wild-type culture was treated with rifampicin for 24 h. The ATP levels were determined. All experiments were performed as biological triplicates. Data are the means ± S.D. Data are analysed with one-way ANOVA; * *p* < 0.05; ** *p* < 0.01.

We examined ATP levels in stationary-phase persister cells after rifampicin treatment and observed that ATP levels in these cells decreased significantly (Figure 9A). We next sought to examine whether Rsh was involved in this phenomenon. ATP levels of ∆*rsh* strain increased significantly compared with wild-type in stationary phase (Figure 9B). As Rsh promoted persister cell formation by regulating *mbcTA* promoter in stationary phase (Figure 8D), we further tested ATP concentrations in ∆*rsh-mbcTA*p strain in stationary phase. ATP levels in ∆*rsh-mbcTA*p strain decreased significantly compared with wild-type or ∆*rsh* backgrounds (Figure 9B). These results suggest that ATP levels in rifampicin-tolerant persister cells are depressed in stationary phase in *B. abortus*, but that these levels increase significantly in stationary phase ∆*rsh* compared with wild-type or C∆*rsh* strains. Moreover, the ATP concentration decreased when *mbcTA* promoter was overexpressed in ∆*rsh* background.

## 3 Discussion

Bacteria escape killing by antibiotics both by genetically-encoded antibiotic resistance and by formation of antibiotic-tolerant persister cells. The latter are one of the major culprits of chronic infections (Bigger, 1944; Nicolau and Lewis, 2022) and are linked to long-term antibiotic treatments that are often required to cure long-lasting infections. Persistence has been observed in diverse species, including *P. aeruginosa* (Grandy et al., 2022), *E. coli* (Chen et al., 2022), *Salmonella* (Hill et al., 2021), *M. tuberculosis* (Sarathy and Dartois, 2020), and *S. aureus* (Vasudevan et al., 2022). Several lines of evidence point to partial bacteria generate high persister cells in clinical patients with chronic infectious symptoms (Mulcahy et al., 2010; Bartell et al., 2020). The (p)ppGpp alarmone, a common regulator of bacterial stringent response, is linked consistently with persistence and virulence (Yan and Bassler, 2019; Zhang et al., 2020). In agreement, we found previously that (p)ppGpp synthetase Rsh contributed to persister cell formation in *B. abortus* in both exponential and stationary phases after rifampicin exposure, but the underlying mechanisms were understood poorly.

Here, we demonstrate that persistence mediated by Rsh is related both to growth phase and to antibiotic class in *B. abortus*. Rsh promotes persistence in the presence of rifampicin and enrofloxacin in different growth phases. Rsh exerted no persister cell effects in late stationary phase with antibiotics other than rifampicin and enrofloxacin, but did promote persistence in exponential phase with doxycycline, polymyxin B, ofloxacin, ampicillin, rifampicin, or enrofloxacin exposure we tested. Rsh is also involved in persistence in *Vibrio splendidus* (Kundra et al., 2020; Li et al., 2021). In contrast with our results, Rsh is not involved in persistence in the presence of ciprofloxacin or gentamicin in stationary phase in *S aureus* (Conlon et al., 2016). Our data indicate that Rsh promotes persister cell formation by a mechanism that may involve rifampicin and enrofloxacin in *B. abortus*. Significantly, rifampicin commonly is used to treat brucellosis (Qureshi et al., 2023). We speculate that Rsh may be a novel therapeutic target in combination with rifampicin for clearing persister cells in chronic *Brucella* infections.

Enrofloxacin belongs to quinolone family that inhibits DNA gyrase which perturbs normal bacterial DNA replication and cell division (Bhatt and Chatterjee, 2022). In contrast, rifampicin inhibits RNA polymerase thereby blocking transcription (Marianelli et al., 2004). The reduced frequency of persister cells in Δ*rsh* mutant is not due to increase antibiotic sensitivity as MIC assays showed no change in susceptibility to rifampicin and enrofloxacin. In addition, Rif target is βsubunit of DNA-dependent RNA polymerase (RNAP) in prokaryotes which entries *M. tuberculosis* via mycobacterial cell wall (Marianelli et al., 2004; Namugenyi et al., 2017). We detected cell envelope permeability of Δ*rsh* mutant using ethidium bromide uptake assays which revealed that mutant displayed increased cell envelope permeability, although the sensitivity of mutant to rifampicin was not different from wild-type. In contrast, Δ*phoY1,* Δ*phoY2,* and Δ*pstA1* mutants of *M. tuberculosis* which possess enhanced cell envelope permeability are hypersusceptible to rifampicin (Shan et al., 2017). The peptidoglycan-associated lipoprotein Omp16 plays an important role in cell wall integrity in *Brucella* (Zhi et al., 2020). The transcript level of *omp16* was unaltered in Rsh mutant compared with wild-type. Thus, our results implicate Rsh promotes persister cell formation in *B. abortus* stationary phase after rifampicin and enrofloxacin exposure, but that this persistence is not due to heightened antibiotic sensitivity, although Δ*rsh* mutant exhibits increased cell envelope permeability.

Bacteria stochastically generate persister cells, but persistence is also associated with environmental parameters that impacts stress signaling pathways including general stress response, SOS response, and stringent response that involves (p)ppGpp. Persister cells are also generated by conditions such as biofilms and hostile host environments (Harms et al., 2016; Peyrusson et al., 2020; Hill et al., 2021). Environmental stresses and host cell milieu are involved in persister cell formation in *B. abortus* (Mode et al., 2022; Liu et al., 2023). Here, we showed altered persistence in the Δ*rsh* mutant during environmental changes including acid exposure, temperature, and phosphate stress. *Brucella* encounters an intracellular acidified environment which may impact persister cell formation inside macrophages (Mode et al., 2022). The Rsh protein did not influence adhesion of *B. abortus* to RAW264.7 macrophage cells, but did impact invasion of these cells. In contrast, adhesion and invasion of a *ΔrelA-ΔspoT* mutant are reduced in *Salmonella pullorum* (Wang et al., 2021). Rsh-dependent persister cell formation is also surveyed during macrophage infection which reveals that the protein is essential for persistence of *B. abortus* in the presence of rifampicin and enrofloxacin in RAW264.7 macrophages. However, Rsh is not involved in persistence during ampicillin exposure in macrophages which is in line with *in vitro* results. The acidified and nutrient-limited intracellular environment in macrophages may be linked to the role of Rsh in persistence. In summary, Rsh is required for persister cell formation under multiple stress signals, as well as in macrophages.

TA modules are linked to the stringent response (p)ppGpp and SOS response (Hall et al., 2017; Ronneau and Helaine, 2019). Moreover, certain type II TA modules are upregulated or activated when (p)ppGpp accumulates in response to nutrient limitation (Hall et al., 2017). In view of the links between Rsh, persistence and stress established here, we examined whether diverse stresses, nutrition starvation and persister cells formation accelerate expression of type II TA modules gene in *B. abortus* stationary phase and whether Rsh modulates type II TA module expression (Ronneau and Helaine, 2019). The *mbcA* was induced under all stresses that were tested (pH, phosphate, NaCl, temperature, oxidative stress) in *B. abortus* which is consistent with previous observations in *M. tuberculosis* (Ariyachaokun et al., 2020). The *BrnT* was also induced under all stress conditions that were tested here, except during exposure to phosphate and H_2_O_2_. In addition, all TA modules were up-regulated during the nutrition starvation. Interestingly, there was no significantly difference during the persister cells formation, except *pem*K. It is possible that the relationship between TA system and them is not obvious during nutrition starvation and persister cells formation. Similarly, certain TAs are induced under these conditions in *E. coli* (Shan et al., 2017). Examination of type II TA module expression in Δ*rsh* mutant in *B. abortus* stationary phase revealed that *brnT* transcription increased and *ratA, mbcA, and relE* decreased compared with wild-type or CΔ*rsh*. As accumulation of the toxin factor in TA systems triggers persistence (Wiradiputra et al., 2022), these results indicate that the mechanism by which Rsh contributes to persister cell formation following rifampicin treatment may be linked with certain type II TA modules including *brnT*, *ratA*, *mbcA*, and *relE*, in *B. abortus* stationary phase.

To further investigate the connections between the (p)ppGpp synthetase Rsh and the *brnTA* and *mbcTA* systems, we used transcriptional fusion plasmids to examine effect of protein on expression of promoters of these TAs in *B. abortus*. The *mbcTA* promoter was active in both wild-type and Δ*rsh* mutant strains and was regulated through Rsh. Furthermore, persister cell levels increased when *mbcTA* promoter was overexpressed in the △*rsh* mutant compared with the △*rsh* mutant in the presence of rifampicin. The *mbcTA locus* is a new RES-Xre type II TA module that is present in many human pathogens including *M. tuberculosis* in which the MbcT toxin reduces cell survival *in vitro* and *in vivo* by promoting secretion of NAD^+^-dependent exotoxins that catalyze NAD^+^ degradation. The bactericidal activity of MbcT is neutralized by its cognate antitoxin MbcA (Freire et al., 2019; Ariyachaokun et al., 2020). In summary, our results show that the (p)ppGpp synthetase Rsh promotes persister cell formation by positive regulation of *mbcTA* after rifampicin exposure in stationary phase. However, whether ppGpp directly or indirectly regulates *mbcTA* needs further investigation.

Due to variation in ATP levels of cells influence persister cells formation through predicting activity of antibiotic targets. Persister formation is accompanied by a drop in intracellular ATP in diverse bacteria, including *S. typhimurium, E. coli* and *S. aureus* (Conlon et al., 2016; Braetz et al., 2017; Shan et al., 2017). Here, ATP levels also decreased in *B. abortus* stationary-phase rifampicin-tolerant persister cells. Although a reduction in ATP levels may partly explain persister cell formation, why some cells have less ATP than other cells remain to be established. Interestingly, the Δ*rsh* mutant in stationary phase exhibited an increase in ATP levels but overexpression of the *mbcTA* promoter in this background reduced ATP levels. Thus, the formation of stationary phase antibiotic-tolerant persister cells in *B. abortus* is associated with a decrease in ATP concentrations. These results also reveal that Rsh promotes rifampicin-tolerant persister cells by regulating *mbcTA* which is accompanied by a reduction in ATP levels in stationary phase in *B. abortus*.

## 4 Conclusion

**Figure 10:**
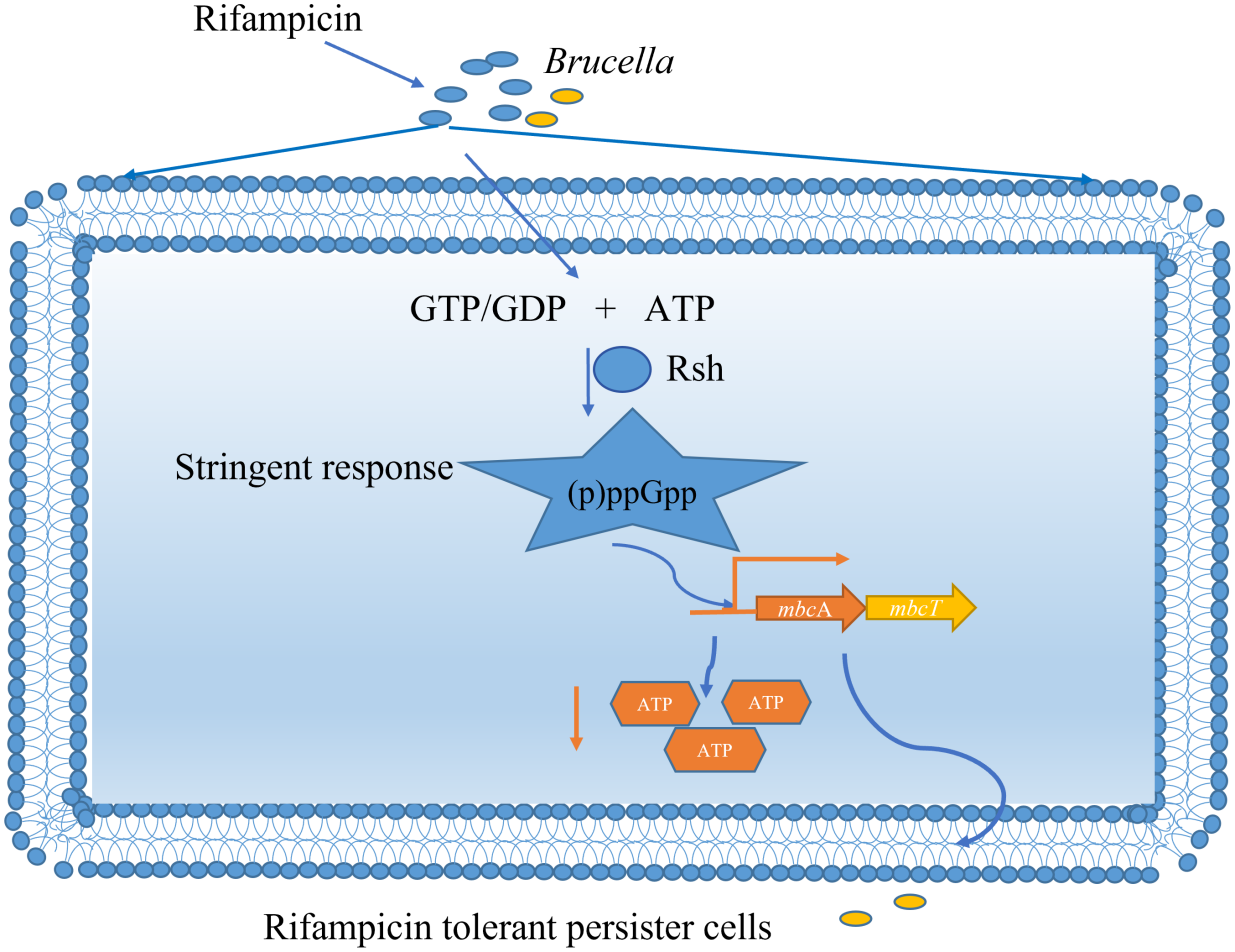
Mechanism of (p)ppGpp synthetase Rsh involving in rifampicin tolerant persister cells formation in *B. abortus* stationary phase. The *B. abortus* forms rifampicin-tolerant persister cells in stationary phase. Stationary-phase rifampicin- tolerant persister cells are controlled by (p)ppGpp synthetase Rsh in *B. abortus.* The Rsh promotes persister cells formation by regulating *mbcAT* promoter and accompanying by decrease of ATP levels in *B. abortus* stationary phase.

We reveal here that persister cells in *B. abortus* is influenced by the (p)ppGpp synthetase Rsh in stationary phase in the presence of rifampicin and enrofloxacin. The impact of Rsh in persister cells is dependent on both antibiotic classes and growth phase. Among the antibiotics that were tested, Rsh promoted persister cell production in the presence of rifampicin and enrofloxacin during different growth phases. We further demonstrate that Rsh promotes persister cells formation by positively regulates *mbcTA* in stationary phase. Finally, positive regulation of *mbcTA* is accompanied by decreased ATP levels after rifampicin exposure in *B. abortus* stationary phase (Figure 10). Overall, our results reveal that (p)ppGpp synthetase Rsh may be used in connection with rifampicin to develop new therapeutic targets and control strategies for preventing brucellosis.

## 5 Materials and methods

### 5.1 Bacterial strains, media, culture conditions, plasmids, and antibiotics

Plasmids and strains are listed in Table 2. The *B. abortus* vaccine strain A19 was supplied by the Shaanxi Veterinary Drug Supervision Institute (shaanxi province) and was cultured on tryptic soy broth (TSB) agar or in liquid medium at 37°C and 180 rpm to mid-exponential phase (12 h), early stationary phase (24 h), or late stationary phase (72 h). *E. coli* was grown in Luria-Bertani (LB) liquid or agar medium at 37°C and 180 rpm. Antibiotics were added when required according to the characteristics of strains and plasmids. Antibiotics used in persister assays were doxycycline, polymyxin B, ofloxacin, ampicillin, rifampin, and enrofloxacin. Antibiotic concentrations used are specified below for individual experiments.

**Table 2:** Strains and plasmids listed in this study.

Plasmids/strains	Description	Reference/source
*E. coli* DH5α	Host strain	Lab stock
*Brucella abortus* A19	Wild type strain	Lab stock
*Δrsh*	*Rsh*: KanR derivative of *B.abortus* A19	Lab stock
*cΔrsh*	AmR: *rsh* complete gene of *B.abortus* A19 Δ*Rsh* strain	Lab stock
pBBR1MCS-5	Gentamicin resistance gene, mod, broad host range cloning vector	Lab stock
pBB-Amp-lacZ	**Ampicillin(Amp) resistance gene, expression vector**	Lab stock
pBB-*mbcT*A*p*-lacZ (mbcTAp)	Amp resistance gene, expression vector	This study
pBB-*brnTAp*-lacZ (*brn*TA*p*)	Amp resistance gene, expression vector	This study
*Δrsh-mbcTAp*	Amp resistance gene, Δ*rsh* carries a pBB-*mbc*A*p*-lacZ	This study
*Δrsh-brnTAp*	Amp resistance gene, Δ*rsh* carries a pBB-*brn*T*p*-lacZ	This study
*WT-brnTAp*	Amp resistance gene, *B. abortus* carries a pBB-*brn*TA*p*-lacZ	This study
*WT-mbcTAp*	Amp resistance gene, *B. abortus* carries a pBB-*mbc*A*p*-lacZ	This study

### 5.2 Strain construction

The promoter fragments of *brnTA* (*brnTA*p) and *mbcTA* (*mbcTA*p) were amplified and cloned into pBB-Amp-*lac*Z expression vector by enzyme digestion and ligation. Plasmid constructs were confirmed by PCR amplification and/or sequencing (Tsingke Biotech Co., Ltd., Beijing, China). The wild-type and Δ*rsh* strains were grown to exponential phase, the cultures were centrifuged, and washed in ddH_2_O to make competent cells. The pBB-*brnTA*p-lacZ and pBB-*mbcTA*p-lacZ plasmids were electro-transformed into wild-type (WT*-brnTA*p, WT-*mbcTAp*) and Δ*rsh* competent cells (Δ*Rsh-mbcTAp*,*ΔRsh-brnTAp*). Strains obtained in this study are summarized in Table 2. All primers are shown in Table 3.

**Table 3:** PCR primers were used in this study.

Primer name	Primer	Sequence (5′-3′)	Amplicon sizes (bp)
*brnT*	*brn*T-F	ATGAAGATCATCTGGGACGAA	90
	*brn*T-R	CAGGAAGAATTCGAAATGCAG	
*ratA*	*rat*A-F	ATGCCTCAATTTACGACCG	111
	*rat*A-R	CAAGGCTTCGCACATAGG	
*mbcA*	*mbc*A-F	ATGAAGCCTGTAATCAGCAAG	128
	mbcA-R	ATCAAGCTTCCATAGATCGG	
*pemK*	*pem*K-F	ATGAAGCGTGGCGAAATATG	121
	*pem*K-R	TGCGCAAATGGTTATCGAG	
*relE*	*rel*E -F	GTGAAGGTTATCGTTTCTCCG	123
	*rel*E-R	ATCACGTTTGAGCCGTT	
*mbcAp*	*mbc*Ap-F	TTATCGCAGCCGAATAAGCC	113
	*mbc*Ap-R	GATCGCCTCCATCTTGTCT	
*mbcAP*	*mbc*AP-F	GGATCCTTCCATACGGCACTTAAAACCC	312
	*mbc*AP-R	CTCGAGGATCGCCTCCATCTTGTCT	
*brnTP*	*brn*TAP-F	GGATCCTTCCAGCATATCCAGCATGAC	343
	*brn*TAP-R	CTCGAGCGTATGTACAATAATTCGTCTGGG	
16srRNA	16srRNA-F	AGAGTTTGATCCTGGCTCAG	123
	16srRNA-R	ATTCCGTAGCAAATGGTACG	
*omp16*	*omp*16-F	ATCCAGTCGATTGCACGTAG	165
	*omp*16-R	GCCGACATTAACGGTGAAGTC	

### 5.3 Persister assays

Strains were grown to late stationary phase (72 h) in 8 mL of fresh TSB liquid medium at 37°C with shaking at 180 rpm. The cultures were plated onto TSB agar and the resulting CFUs were used as initial values. Doxycycline (1.2 μg/mL), polymyxin B (40 μg/mL), ofloxacin (40 μg/mL), ampicillin (8 μg/mL), rifampicin (40 μg/mL), or enrofloxacin (12 μg/mL) were added and samples were removed at 12 h, 24 h, and 36 h, and centrifuged at 6, 000 × *g* for 10 min. The pellets were collected and washed once in PBS and then diluted in PBS for viable counts.

In other experiments, bacteria were grown to exponential phase (12 h), (24 h) or late stationary phase (72 h) in 8 mL of fresh TSB liquid medium at 37°C with shaking at 180 rpm. Antibiotics were added as described above and incubation continued for 36 h. Samples before and after antibiotic treatment were collected by centrifugation at 6, 000 × *g* for 10 min. Cell pellets were washed once in PBS and then diluted in PBS for viable counts on TSB agar plates. The surviving colonies were determined in treated and untreated samples after 72 h of incubation at 37°C.

For adhesion assays, bacteria were incubated with macrophage cells for 1 h at 37°C in 5% CO_2_ atmosphere. Infected cells were washed once in PBS (pH 7.2), collected, and then lysed with 0.1% Triton X-100. The lysates were removed and 10-fold serial dilutions were made with PBS to assess viable counts as the initial time point. The lysates were centrifuged, collected, and resuspended in TSB containing ampicillin (4 μg/mL), rifampicin (20 μg/mL), or enrofloxacin (6 μg/mL) for detection of intracellular persister cell formation at 2 h, 6 h, and 24 h. Samples were collected, washed once in PBS, and then 10-fold serially diluted in PBS for viable counts. The dilutions were plated onto TSB agar plates and incubated at 37°C for 72 h.

### 5.4 MIC assays

The MICs of wild-type *B. abortus* A19 and the Δ*rsh* and *C*Δ*rsh* derivatives on rifampicin and enrofloxacin were determined as previously reported using two-fold serial dilutions of antibiotics with TSB broth (Liu et al., 2023). The strains were grown to OD_600_ = 0.6 in TSB broth and diluted to OD_600_ = 0.01 in 5 mL fresh medium. Antibiotics were dispensed in 96-well polyethylene plates and cultures were added. Cultures without antibiotics served as controls. The cultures were incubated at 37°C for 36–48 h. The MIC was taken as the minimum concentration of antibiotic that significantly inhibited growth of bacteria relative to that of the positive control.

### 5.5 Stress assays

Strains were cultured to stationary phase in TSB medium at 37°C with shaking for 72 h. Stationary phase cultures were adjusted to 1–2 × 10^8^ CFU/mL. The cultures were exposed to different stress conditions for 1 h. For acid stress, cultures were incubated in TSB medium with the addition of HCl (pH 4.5). Phosphate starvation involved incubation in 0.01 M PBS (pH 7.0). For NaCl stress, the cultures were incubated in TSB medium with the addition of 2% NaCl. Temperature stress involved growth in TSB medium at 25°C whereas oxidative stress involved growing in TSB containing H_2_O_2_ (1 mM). For nutrition starvation, the cultures were harvested and incubated in PBS with with 0.05% Tween 80 for 4 h (Li et al., 2022). Untreated cultures in TSB medium were used as controls. The cultures were centrifuged, washed once in PBS, and the cell pellets were resuspended in TSB liquid medium containing rifampicin (40 μg/mL) with shaking at 37°C for 24 h for detection of persister formation. Persister cell assays were conducted as outlined above.

### 5.6 Cell culture and infection

Adhesion and invasion assays were performed using macrophage cell line RAW264.7 (National Collection of Authenticated Cell Cultures). Briefly, bacterial strains were grown in TSB medium at 37°C with shaking for 72 h. The cultures were centrifuged at 6,000 × *g* for 10 min at 4°C, washed three times with PBS, and resuspended in Dulbecco’s modified Eagle medium (DMEM; Hyclone, United States). RAW264.7 cells were cultured in DMEM with 10% unheated fetal bovine serum (FBS) using 12-well culture plates at a density of 8 × 10^5^ cells per well. Stationary phase bacterial cultures were added at a multiplicity of infection of 200. Infected cells were centrifuged at 100 × *g* for 5 min. The adherence of bacteria to macrophages was continued for 1 h at 37°C in 5% CO_2_ atmosphere. Infected cells were washed once in PBS (pH 7.2), collected, and lysed with 0.1% Triton X-100. The lysates were removed for 10-fold serial dilutions in PBS for viable counts. Invasion of *B. abortus* in macrophage cells was performed by adding gentamycin (50 μg/mL) to medium and incubating for an additional 1 h at 37°C in 5% CO_2_ atmosphere. Infected cells also were lysed with 0.1% Triton X-100 for viable counts. Dilutions were plated onto TSB agar plates and incubated at 37°C for 72 h.

### 5.7 Ethidium bromide uptake assays

Ethidium bromide uptake was tested as described previously with some minor modifications (Namugenyi et al., 2017). *B. abortus* strains were grown to stationary phase (72 h). The cultures were centrifuged, washed once with PBS, and resuspended in PBS to OD_600_ = 0.6–0.8. Ethidium bromide was added to a final concentration of 2 μg/mL. Uptake was measured using 96-well microplates with excitation at 544 nm and emission at 590 nm. The uptake rates were calculated using data in the linear range between 0 and 30 min. Each experiment was repeated at least three times.

### 5.8 qRT-PCR

Total RNA was extracted from stationary-phase cultures of *B. abortus* after 72 h of growth using Trizol (Takara, Bio, Dalian, China) by conventional methods. The cDNA samples were generated with the PrimeScript™ RT reagent Kit (Takara) according to the manufacturer’s instructions. Real-time quantitative primers are listed in Table 3. Quantitative real-time PCRs (qRT-PCR) was performed using SYBR^®^ green enzyme (Vazyme, Nanjing, China). Fold change was calculated by the 2^−ΔΔCt^ method. Target genes were normalized internally to 16S rRNA.

### 5.9 β-Galactosidase activity assays

In brief, the pBB-*brnTA*p-*lacZ* and pBB-*mbcTA*p-*lacZ* report plasmids were transformed into wild-type and ∆*rsh* competent cells. Single colonies were selected and cultured to stationary phase. The cultures were collected and washed twice with cold PBS. 100 μL of bacterial suspensions were added to 2 mL EP tubes. 600 μL of pre-cooled Z buffer (100 mM Na_2_HPO_4_, 40 mM NaH_2_PO_4_, 10 mM KCl, 1 mM MgSO_4_, 5.4 μL of β-mercaptoethanol, pH 7.5) were added to permeabilize bacteria. 200 μL of resuspended cultures were used to determine OD_600_ values. 50 μL of 0.1% SDS and 100 μL of chloroform were added. The samples were mixed and incubated at room temperature for 5 min. O-nitrophenyl-β-D-galactoside (ONPG) (200 μL of 4 mg/mL) was added and incubated at room temperature for 10 min. 1 M Na_2_CO_3_ (500 μL) was added to terminate the reactions. Samples were centrifuged at 12,000 × g for 5 min and 200 μL of supernatants were collected to determine OD_420_, OD_550_, and OD_600_ values. Miller units were calculated using the formula: Miller units = 1,000 × (OD_420_–1.75 × OD_550_)/T (min) × V (mL) × OD_600_. In addition, stationary phase cultures were diluted 10-fold and spotted onto TSB agar plates containing X-gal (2 mg/mL) and incubated at 37°C. Colony color was observed.

### 5.10 ATP assays

ATP levels of persister cells in stationary phase and stationary cultures were measured using an ATP assay kit (Beyotime Biotechnology, Shanghai, China) according to the manufacturer’s instructions.

### 5.11 Statistical analysis

All experiments were performed as three biological replicates. GraphPad Prism 8.0 (San Diego, CA, USA) was used for data graphing. Data were analysed with one-way or two-way analysis of variance (ANOVA) using SPSS 22.0 (IBM, Chicago, Ill, USA). *p* > 0.05 (ns) was considered not significant. *p* < 0.05 (*) was considered significant. *p* < 0.01 (**) was considered highly significant. Data points indicate the mean values of the results of at least three biological replicates and error bars indicate standard errors of the mean.

## Data availability statement

The original contributions presented in the study are included in the article/Supplementary material, further inquiries can be directed to the corresponding author.

## Author contributions

XL: Data curation, Formal analysis, Methodology, Project administration, Software, Supervision, Validation, Writing – original draft, Writing – review & editing. PW: Data curation, Formal analysis, Methodology, Supervision, Writing – review & editing. NY: Data curation, Software, Writing – review & editing. YZ: Formal analysis, Methodology, Software, Writing – review & editing. YY: Methodology, Software, Writing – review & editing. MH: Methodology, Writing – review & editing. MZ: Methodology, Software, Writing – review & editing. DZ: Investigation, Writing – review & editing. WL: Methodology, Writing – review & editing. YJ: Formal analysis, Methodology, Supervision, Writing – review & editing. AW: Formal analysis, Funding acquisition, Resources, Supervision, Writing – review & editing.
