# Supplementary material for: The (p)ppGpp synthetase Rsh promotes rifampicin tolerant persister cell formation in Brucella abortus by regulating the type II toxin-antitoxin module mbcTA
Source: Front Microbiol. 2024 May 22;15:1395504. doi: 10.3389/fmicb.2024.1395504 (PMC11150624; doi:10.3389/fmicb.2024.1395504)
Supplement: Supplementary file 1 [file Data_Sheet_1.docx]

**Supplementary figure 1 The PCR amplification of *mbcT*A and *brnTA* promoter of *B. abortus* A19.** Lane M. DNA marker; 1 *mbcTA* promoter; 2 *mbcT*A promoter; 3 *brnTA* promoter*;* 4 *brnTA* promoter
